# Effect of urine alkalization on urinary inflammatory markers in cystinuric patients

**DOI:** 10.1093/ckj/sfae040

**Published:** 2024-02-22

**Authors:** Caroline Prot-Bertoye, Vincent Jung, Isabelle Tostivint, Kevin Roger, Jean-François Benoist, Anne-Sophie Jannot, Alexis Van Straaten, Bertrand Knebelmann, Ida Chiara Guerrera, Marie Courbebaisse

**Affiliations:** Assistance Publique-Hôpitaux de Paris, Hôpital Européen Georges Pompidou, Service de Physiologie – Explorations fonctionnelles, Paris, France; Centre de Recherche des Cordeliers, INSERM, Sorbonne Université, Université Paris Cité, Paris, France; CNRS ERL 8228 – Laboratoire de Physiologie Rénale et Tubulopathies, Paris, France; Centre de Référence des Maladies Rénales Héréditaires de l'Enfant et de l'Adulte (MARHEA), Paris, France; Centre de Référence des Maladies Rares du Calcium et du Phosphate, Paris, France; Association LUNNE Lithiases UriNaires Network, Paris, France; Proteomics Platform Necker, Université Paris Cité – Structure Fédérative de Recherche Necker, INSERM US24/CNRS UAR3633, Paris, France; Centre de Référence des Maladies Rénales Héréditaires de l'Enfant et de l'Adulte (MARHEA), Paris, France; Association LUNNE Lithiases UriNaires Network, Paris, France; Assistance Publique-Hôpitaux de Paris, Hôpital de la Pitié Salpêtrière, Service de Néphrologie, Paris, France; GRC 20 ARDELURO groupe de recherche clinique Analyse, Recherche, Développement et Evaluation en Endourologie et Lithiase Urinaire, Médecine Sorbonne Université, Paris, France; Proteomics Platform Necker, Université Paris Cité – Structure Fédérative de Recherche Necker, INSERM US24/CNRS UAR3633, Paris, France; Faculté de pharmacie, Université Paris Saclay, Orsay, France; Assistance Publique-Hôpitaux de Paris, Hôpital Necker, Service de Biochimie métabolique, Paris, France; Assistance Publique-Hôpitaux de Paris – Centre, Université Paris Cité, Hôpital Européen Georges Pompidou, Service d'informatique Médicale, Santé Publique et Biostatistiques, Paris, France. HeKA, Centre de recherche des Cordeliers, INSERM, INRIA, Paris, France; Assistance Publique-Hôpitaux de Paris – Centre, Université Paris Cité, Hôpital Européen Georges Pompidou, Service d'informatique Médicale, Santé Publique et Biostatistiques, Paris, France. HeKA, Centre de recherche des Cordeliers, INSERM, INRIA, Paris, France; Faculté de médecine, Université Paris Cité, Paris, France; Assistance Publique-Hôpitaux de Paris, Hôpital Necker, Service de Néphrologie, Paris, France; INEM Unité Inserm U1151, Paris, France; Proteomics Platform Necker, Université Paris Cité – Structure Fédérative de Recherche Necker, INSERM US24/CNRS UAR3633, Paris, France; Assistance Publique-Hôpitaux de Paris, Hôpital Européen Georges Pompidou, Service de Physiologie – Explorations fonctionnelles, Paris, France; Centre de Référence des Maladies Rénales Héréditaires de l'Enfant et de l'Adulte (MARHEA), Paris, France; Centre de Référence des Maladies Rares du Calcium et du Phosphate, Paris, France; Association LUNNE Lithiases UriNaires Network, Paris, France; Faculté de médecine, Université Paris Cité, Paris, France; INEM Unité Inserm U1151, Paris, France

**Keywords:** cystinuria, inflammation, nephrolithiasis, urine proteomics

## Abstract

**Background:**

Cystinuria is associated with a high prevalence of chronic kidney disease (CKD). We previously described a urinary inflammatory-protein signature (UIS), including 38 upregulated proteins, in cystinuric patients (Cys-patients), compared with healthy controls (HC). This UIS was higher in Cys-patients with CKD. In the present observational study, we aimed to investigate the UIS in Cys-patients without CKD and patients with calcium nephrolithiasis (Lith-patients), versus HC and the effect of urine alkalization on the UIS of Cys-patients.

**Methods:**

UIS was evaluated by nano-liquid chromatography coupled to high-resolution mass spectrometry in adult HC, Lith-patients and non-treated Cys-patients with an estimated glomerular filtration rate >60 mL/min/1.73 m^2^, and after a 3-month conventional alkalizing treatment in Cys-patients.

**Results:**

Twenty-one Cys-patients [12 men, median age (interquartile range) 30.0 (25.0–44.0) years], 12 Lith-patients [8 men, 46.2 (39.5–54.2) years] and 7 HC [2 men, 43.1 (31.0–53.9) years] were included. Among the 38 proteins upregulated in our previous work, 11 proteins were also upregulated in Cys-patients compared with HC in this study (5 circulating inflammatory proteins and 6 neutrophil-derived proteins). This UIS was also found in some Lith-patients. Using this UIS, we identified two subclusters of Cys-patients (5 with a very high/high UIS and 16 with a moderate/low UIS). In the Cys-patients with very high/high UIS, urine alkalization induced a significant decrease in urinary neutrophil-derived proteins.

**Conclusion:**

A high UIS is present in some Cys-patients without CKD and decreases under alkalizing treatment. This UIS could be a prognostic marker to predict the evolution towards CKD in cystinuria.

KEY LEARNING POINTS **What was known:**

- Cystinuria is associated to a high prevalence of chronic kidney disease (CKD).
- It is currently difficult to predict the progression of kidney damage.

**This study adds:**

- Using proteomics, this paper describes for the first time a urinary inflammatory-protein signature (UIS) including circulating inflammatory proteins and neutrophil-derived proteins, present in some cystinuric patients without CKD.
- In cystinuric patients with a high UIS, urinary neutrophil-derived proteins decrease under 3-month alkalization, the cornerstone of the preventive medical treatment.

**Potential impact:**

- This UIS could be a specific marker of renal inflammation and a new prognostic marker to predict the evolution towards CKD in cystinuria.

## INTRODUCTION

Cystinuria (OMIM 220100) is the most frequent monogenic cause of renal calculi and is responsible for 1% and 4%–8% of nephrolithiasis in adults and children, respectively [1–4]. Cystinuria affects dibasic amino acid and cystine reabsorption in the proximal tubule. Because of its poor solubility at a typical urine pH <7, excess cystine results in urinary cystine stone recurrent formation. Alkaline hyperdiuresis is the cornerstone of the medical treatment with a goal for urinary pH (_U_pH) between 7.5 and 8 [4, 5]. Most patients have AA or BB genotypes, which denotes two mutated alleles in *SLC3A1* (encoding the Neutral and Basic Amino Acid Transport protein rBAT), or *SLC7A9* (encoding subunit b0,+AT of this transporter), respectively [4].

Chronic kidney disease (CKD) is more prevalent in cystinuric patients (Cys-patients) than in patients with other kinds of stones [6–8] or in the general population [9]. In a large French cohort of 314 Cys-patients aged ≥16 years, we showed that 27% had impaired renal function defined by an estimated glomerular filtration rate (eGFR) <60 mL/min/1.73 m^2^ [9]. After exclusion of known risk factors for CKD, CKD prevalence remained higher in Cys-patients than in the general population [9], suggesting that other risk factors for CKD due to cystinuria itself may exist, such as renal inflammation. It is currently difficult to predict the evolution of kidney damage and genetics is poorly correlated to the clinical course of cystinuria [2, 4].

In accordance with this hypothesis, we have described the presence of a urinary inflammatory-protein signature (UIS) in Cys-patients by analyzing the urinary proteomic pattern of 8 patients and 10 healthy controls by mass spectrometry [10]. A panel of 165 proteins was identified that were significantly different between both groups, of which 38 were overexpressed in Cys-patients and discriminated between those patients and controls, and further discriminated between severe and moderate forms of the disease (differentiated according to an eGFR <60 or ≥60 mL/min/1.73 m^2^).

These data were very innovative in this pathology but it was also important to study the UIS of Cys-patients without CKD. Indeed, this UIS may be an early prognostic biomarker, provided we can identify it before the occurrence of CKD. Moreover, the effect of urine alkalization on urinary inflammatory proteins expression deserves to be tested.

The objectives of the present study are: (i) to study the urinary inflammatory protein profile by mass spectrometry in Cys-patients without CKD and in patients with nephrolithiasis of other origin (versus healthy controls); and (ii) to study the effect of urine alkalization on the UIS of Cys-patients. For the purpose of the present study, we focused on the overexpression of urinary inflammatory proteins among those that have been highlighted in our previous work [10].

## MATERIALS AND METHODS

### Study population

We conducted an observational study in three centers (European Georges Pompidou, Necker-Enfants Malades and la Pitié-Salpétrière hospitals; Assistance Publique-Hôpitaux de Paris, France). Inclusion criteria were the following: ≥18 years of age, proven diagnosis of either cystinuria (Cys-patients) or of non-cystinuric nephrolithiasis (Lith-patients), GFR estimated by Chronic Kidney Disease Epidemiology Collaboration (CKD-EPI) >60 mL/min/1.73 m^2^, sterile urine, patient able to understand the information note and having signed the written informed consent, and patient affiliated to a social security scheme. Cys-patients could not be included if they received a cysteine-binding thiol treatment or an alkalizing treatment (citrate or bicarbonate salts) during the 3 months before inclusion. No treatment wash-out was allowed to include a patient.

If the last renal imaging dated back more than 1 month, a renal and bladder ultrasound to look for stones was performed within the 10 days following inclusion.

Healthy controls (HC), aged ≥18 years, with no history of CKD or nephrolithiasis, no urinary crystal and no bacteriuria, were also included. Patients and HC were included between April 2015 and July 2019, and data were collected between April 2015 and December 2019.

Oral and written information was provided to patients and each patient signed written informed consent. The study received approval from the advisory committee on information processing research in the field of health (CCTIRS number 16-830) and was registered in ClinicalTrials.gov Identifier (NCT03836144) in February 2019. The study complies with the Declaration of Helsinki.

### Alkalizing treatment for cystinuric patients

An alkalization protocol was given to the physician in charge of the patient: the initial dosage of potassium citrate was 4 g/day divided into three to four intakes. If the _U_pH target (7.5–8) [4] was not reached after the first 2 weeks, the dose was increased by 2 g. If the _U_pH remained below 7.5 after 2 weeks with 6 g of potassium citrate per day, sodium bicarbonate in the form of alkalizing water or officinal preparation was added. The dosage of alkalizing treatment was adjusted according to the _U_pH, which was self-monitored by patients using urinary sticks. In case of poor digestive tolerance, the dosage of potassium citrate was reduced by 2 g per day and sodium bicarbonate could be added.

The monitoring of blood electrolytes under treatment was performed according to the habits of each physician.

For Cys-patients, a renal ultrasound or computed tomography scan was performed and the blood and urine samples described below were collected a first time at the basal state (without treatment) and after 3 months of alkalizing treatment.

### Biological analyses

Blood electrolytes, creatinine to estimate GFR by CKD-EPI, complete blood count and C-reactive protein (CRP), cytobacteriological urine exam, urine electrolytes and creatinine, microalbuminuria and proteinuria were analyzed using the local standard methods. For crystalluria, well-homogenized spot urine specimens set in a Malassez cell were examined under a light microscopy equipped with a polarized light device. The type of crystal was determined based on the morphology, coupled with their polarizing features. _U_pH was measured in spot urine samples using urinary sticks.

For proteomic analysis, a spot urine sample was collected and protease inhibitors [10] were added immediately after voiding. All samples were frozen within 2 h from collection, stored in a centralized monitored freezer at −80°C and processed by the same operator. Nano LC-MS/MS protein identification and quantification are described in the Supplementary data.

### Statistical analyses

Baseline characteristics were compared between groups using the Wilcoxon test and Kruskal–Wallis test for continuous variables and the Chi-squared test or Fisher’s exact test for qualitative variables. For comparisons between the two visits, paired tests were used, paired Wilcoxon test for quantitative variables and McNemar test for qualitative variables were calculated. Statistical analyses were performed using R statistical software version 4.1.3. Statistical analysis regarding Nano LC-MS/MS protein identification and quantification are described in the Supplementary data.

## RESULTS

### Description of participants

We included 21 cystinuric patients (Cys0-patients), 12 Lith-patients (all having common calcium nephrolithiasis) and 7 HC. Twenty-three participants were excluded or screened and non-included due to an eGFR <60 mL/min/1.73 m^2^ or bacteriuria or, for HC, the presence of urinary crystals. At inclusion, the three groups had similar characteristics except for age, which was younger, and _U_pH, which was higher, in the Cys0-patients, and hematuria and presence of urinary crystals which were more frequent in Cys0-patients and Lith-patients (Table 1). At inclusion, compared with Lith-patients, Cys0-patients were significantly younger, had an earlier onset of symptoms, less frequently had diabetes, had a higher _U_pH and more frequently had urinary crystals (Supplementary data, Table S1).

**Table 1:** Baseline characteristics of the three groups.

		Cys0-patients (*N* = 21)	Lith-patients (*N* = 12)	HC group (*N* = 7)	*P*-value
Age (years)		29.9 (24.6; 42.8)	46.5 (39.5; 54.2)	43.1 (31.5; 50.8)	.044
Sex	W	9 (42.9)	4 (33.3)	5 (71.4)	.329
	M	12 (57.1)	8 (66.7)	2 (28.6)	
BMI (kg/m^2^)		24.4 (24.1; 29)	26.35 (22.7; 28.9)	22.8 (20.7; 23.7)	.087
eGFR (mL/min/1.73 m^2^)		95 (88; 111)	106 (97.5; 117.7)	116 (91; 118.5)	.243
Blood leucocytes (10^3^/mm^3^)		5.95 (5.2; 7.6)	6.3 (5.1; 7.2)	6.6 (5.6; 7.5)	.923
CRP >5 mg/L	N	17 (85)	11 (91.7)	7 (100)	.806
	Y	3 (15)	1 (8.3)	0 (0)	
	NA	1	0	0	
U pH		6.65 (6; 7)	5.9 (5.4; 6)	5.6 (5.4; 6.2)	.004
	NA	1	1	0	
U specific gravity		1015 (1015; 1020)	1012 (1006.5; 1020)	1015 (1015; 1022.5)	.518
	NA	3	1	1	
U leucocytes ≥10/mm^3^	N	13 (61.9)	7 (58.3)	7 (100)	.133
	Y	8 (38.1)	5 (41.7)	0 (0)	
U red blood cells ≥10/mm^3^	N	10 (47.6)	7 (58.3)	7 (100)	.04
	Y	11 (52.4)	5 (41.7)	0 (0)	
U protein/creatinine ratio>30 mg/mmol	N	20 (95.2)	11 (91.7)	7 (100)	1
	Y	1 (4.8)	1 (8.3)	0 (0)	
U crystals^a^	N	5 (23.8)	7 (58.3)	7 (100)	.001
	Y	16 (76.2)	5 (41.7)	0 (0)	

Baseline characteristics were described using median (interquartile range) for continuous variables and *n* (%) for qualitative variables.

^a^Urinary crystals could be crystals of cystine or other types of crystals.

M: men; W: women; N: no; Y: yes; NA: missing data; U: urinary; BMI: body mass index.

### Differential expression of urinary proteins between Cys0-patients and HC

**Figure 1:**
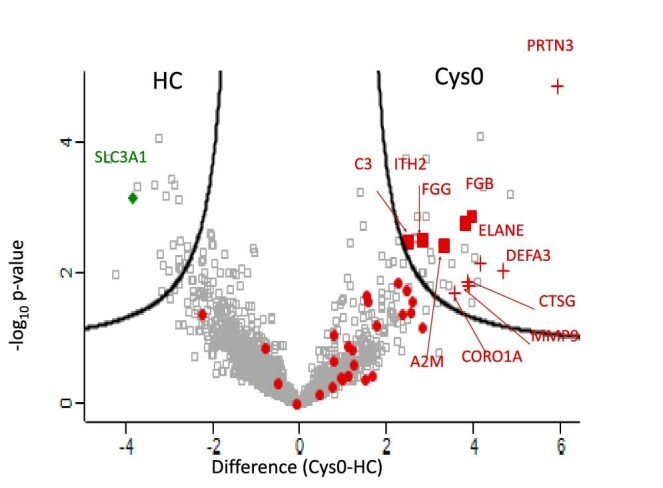
Differential proteomes of cystinuric patients at baseline (Cys0, *n* = 21) versus HC (*n* = 7). The proteome of the 21 Cys0-patients was compared with that of the 7 HC. Volcano plot representing the statistical comparison of the protein Label Free Quantification intensities of the Cys0 group versus the HC group. Volcano plot was established using S0 = 1 and False Discovery Rates = 0.1. The abscissa reports the fold-change in logarithmic scale (difference), the ordinate the –log_10_ *p*-value. Neutrophil-derived proteins and inflammatory circulating proteins were highlighted in red crosses and red filled scares, respectively. The protein rBAT encoded by the *SLC3A1* gene is highlighted with a green diamond. For the name of the protein corresponding to the gene name, refer to Supplementary data, Table S2.

Among the 38 urinary proteins upregulated in cystinuric patients versus HC in our previous work [10], 11 were also upregulated in Cys0-patients compared with HC in this study (Fig. 1). These 11 proteins were either neutrophil-derived proteins or inflammatory circulating proteins (Supplementary data, Table S2) and were considered as being the new UIS of cystinuria patients without CKD. Most of the remaining 27 proteins were also found increased, although not significantly, in Cys0-patients compared with HC. The abundance of rBAT protein was significantly higher in HC than in Cys0-patients with no significant difference between AA (*n* = 14) and BB (*n* = 6) Cys-patients (data not shown).

### Differential clustering between Cys0-patients, Lith-patients and HC according to the UIS

**Figure 2:**
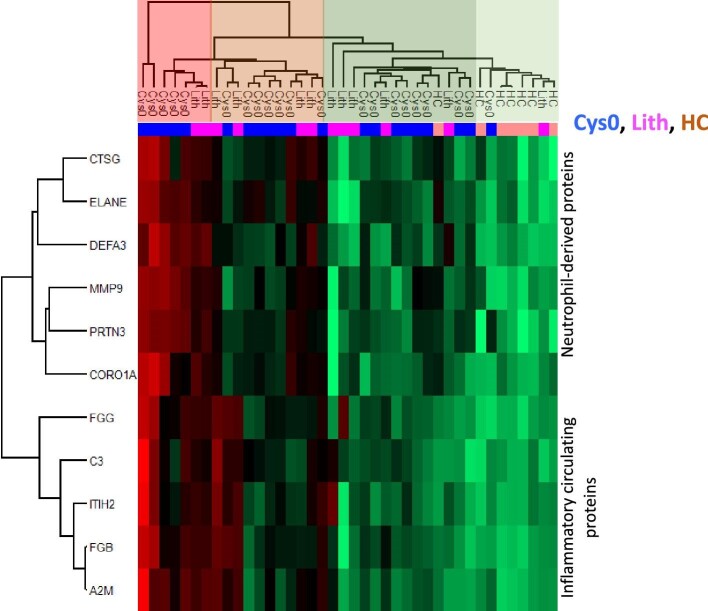
Heatmap of the proteins involved in the UIS among cystinuric patients at baseline (Cys0, *n* = 21, in blue), patients with other types of nephrolithiasis (Lith, *n* = 12, in pink) and HC (*n* = 7, in orange). The heatmap representation was performed on Perseus software (version 1.6.15.0) on z-scored Label Free Quantification intensity of the proteins with a green–red color gradient where red color represents highly abundant proteins and green low abundant proteins. Two clusters of proteins were highlighted corresponding to neutrophil-derived proteins and inflammatory circulating proteins. Moreover, individuals were clustered in four groups corresponding to different level of UIS: “strong” (red), “moderate” (orange), “low” (dark green) and “no” (light green). For the name of the protein corresponding to the gene name, refer to Supplementary data, Table S2.

Using this new UIS composed of the 11 most deregulated proteins, we could highlight four main clusters of participants according to hierarchical clustering using Euclidean distances: (i) very high (*n* = 2) or high (*n* = 5) UIS, (ii) moderate UIS (*n* = 11), (iii) low UIS (*n* = 14), and (iv) without UIS (*n* = 8) (Fig. 2). In the three first clusters (very high/high, moderate or low UIS), Cys0-patients and Lith-patients were equally represented and HC were underrepresented, whereas the cluster with no UIS was composed of more than 85% of the HC and of less than 10% of Cys0- or Lith-patients (Supplementary data, Table S3). The subcluster with the very high UIS was, however, exclusively composed of Cys0-patients (*n* = 2). In line with this result, the intensity of expression of each of the 11 proteins constituting the UIS was not different between Cys0- and Lith-patients. However, this expression was significantly higher, for some proteins (all inflammatory circulating proteins and, among neutrophil-derived proteins, proteinase 3 and neutrophil defensing), in Cys0- and Lith-patients compared with HC (Supplementary data, Fig. S1). The expression of neutrophil elastase, cathepsin G and matrix metalloproteinase-9 was higher in Cys0-patients versus HC, but not in Lith-patients versus HC.

### Stratification of cystinuric patients according to the UIS

**Figure 3:**
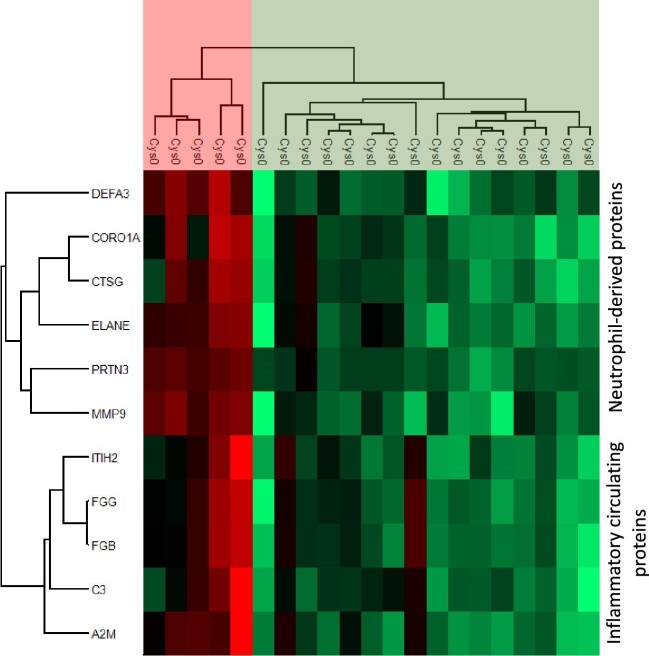
Heatmap of the proteins involved in the UIS among the 21 cystinuric patients at baseline (Cys0). The heatmap representation was performed on Perseus software (version 1.6.15.0) on z-scored Label Free Quantification intensity of the proteins with a green–red color gradient where red color represents highly abundant proteins and green low abundant proteins. Two clusters of proteins were highlighted corresponding to neutrophil-derived proteins and inflammatory circulating proteins. Moreover, individuals were clustered in two groups corresponding to different level of UIS: 5 patients had a very high or high inflammatory urinary signature (red) and 16 patients had a moderate or low UIS (green). For the name of the protein corresponding to the gene name, refer to Supplementary data, Table S2.

This UIS also allowed us to stratify Cys0-patients in two main clusters: 5 patients had a very high (*n* = 2) or high (*n* = 3) UIS, whereas 16 patients had a moderate (*n* = 8) or low (*n* = 8) UIS (Fig. 3). Compared with the 16 Cys0-patients with a moderate/low UIS, the 5 Cys0-patients with a very high/high UIS had an earlier onset of symptoms and more frequently had significant aseptic leukocyturia, but less frequently had urinary cystine crystals (Table 2). The intensity of expression of each of the 11 proteins constituting the UIS was not different between AA (*n* = 14) and BB (*n* = 6) Cys-patients (Supplementary data, Fig. S2).

**Table 2:** Baseline characteristics of patients with cystinuria according to the intensity of the UIS.

		Moderate/low UIS (*N* = 16)	Very high/high UIS (*N* = 5)	*P*-value
Age (years)		31.45 (26.1; 43.3)	24.15 (20.6; 29.9)	.153
Sex	W	5 (31.2)	4 (80)	.119
	M	11 (68.7)	1 (20)	
BMI (kg/m^2^)		24.45 (23.9; 28.8)	24.4 (24.4; 29.8)	.71
Age at first symptoms (years)		17.5 (15.75; 25)	13 (7; 16)	.047
Diabetes mellitus	N	16 (100)	5 (100)	
High blood pressure	N	15 (93.7)	5 (100)	1
	Y	1 (6.2)	0 (0)	
Other kidney disease	N	15 (93.7)	5 (100)	1
	Y	1 (6.2)	0 (0)	
Genotype	AA	10	4	1
	BB	5	1	
	NA	1		
eGFR (mL/min/1.73 m^2^)		95 (87.2; 107)	91 (90; 111)	.836
Blood leucocytes (10^3^/mm^3^)		5.93 (5.2; 7.7)	6.94 (5.7; 7.1)	.773
CRP >5 mg/mL	N	13 (81.2)	4 (100)	1
	Y	3 (18.7)	0 (0)	
	NA	0	1	
Renin–angiotensin inhibitors treatment	N	15 (93.7)	5 (100)	1
	Y	1 (6.2)	0 (0)	
Anti-inflammatory treatment^a^	N	15 (100)	3 (75)	.211
	Y	0 (0)	1 (25)	
	NA	1	1	
Urinary tract infection^a^	N	14 (87.5)	4 (80)	1
	Y	2 (12.5)	1 (20)	
Spontaneous expulsion of stones^a^	N	10 (66.7)	2 (50)	.603
	Y	5 (33.3)	2 (50)	
	NA	1	1	
Urological intervention^a^	N	12 (75)	4 (80)	1
	Y	4 (25)	1 (20)	
U specific gravity		1020 (1015; 1020)	1012.5 (1008.7; 1015)	.051
	NA	2	1	
U pH		6.8 (6; 7)	6.5 (6; 7)	.535
	NA	1	0	
U cystine crystals	N	4 (25)	4 (80)	.047
	Y	12 (75)	1 (20)	
Stones^b^	N	5 (33.3)	0 (0)	.266
	Y	10 (66.7)	5 (100)	
	NA	1	0	
U leucocytes >10/mm^3^	N	12 (75)	1 (20)	.047
	Y	4 (25)	4 (80)	
U red blood cells >10/mm^3^	N	9 (56.2)	1 (20)	.311
	Y	7 (43.7)	4 (80)	
U protein/creatinine ratio >30 mg/mmol	N	16 (100)	4 (80)	.238
	Y	0 (0)	1 (20)	

Baseline characteristics were described using median (interquartile range) for continuous variables and *n* (%) for qualitative variables.

^a^In the 6 months prior to Visit 1.

^b^Presence of calculi in the renal cavities or in the urinary tract.

M: men; W: women; N: no; Y: yes; NA: missing data; U: urinary; BMI: body mass index.

### Effect of urine alkalization on the UIS in cystinuric patients

**Figure 4:**
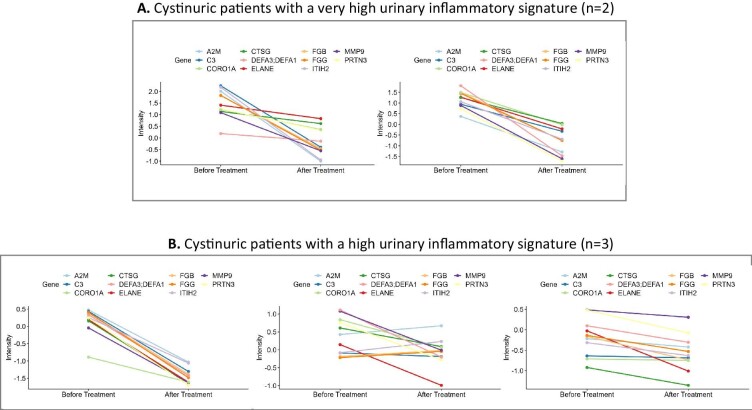
Profile plots of the neutrophil-derived proteins and inflammatory circulating proteins in the 5 cystinuric patients showing a very high (*n* = 2) (**A**), or high (*n* = 3) (**B**) UIS before and after the 3-month alkalizing treatment. For the name of the protein corresponding to the gene name, refer to Supplementary data, Table S2.

Among the 21 Cys0-patients, 16 (Cys1-patients) were explored before and after a 3-month alkalizing treatment. The Cys1-patients and the five patients lost to follow-up after the first visit had similar baseline characteristics, except for a significantly lower eGFR in Cys1-patients (Supplementary data, Table S4). _U_pH significantly increased and hematuria frequency significantly decreased after the 3-month treatment (Table 3). The percentage of Cys1-patients with leukocyturia was similar before and after the 3-month alkalizing treatment. Among the Cys1-patients, 5 had a very high/high and 11 had a moderate/low baseline UIS. Among the 11 patients with a moderate/low baseline UIS, the alkalizing treatment had no effect on the intensity of the UIS (data not shown), whereas this treatment decreased the intensity of the UIS in the 5 cystinuric patients with a very high/high baseline UIS (Fig. 4). When looking at protein per protein, we observed a significant or nearly significant decrease in all neutrophil-derived proteins after the 3-month treatment and a less pronounced decrease in the urinary inflammatory circulating proteins, with a reduction close to significance only for C3 and fibrinogen gamma and beta chains (Supplementary data, Fig. S3).

**Table 3:** Evolution of biochemical parameters in cystinuric patients after 3 months of alkalizing treatment.

		Visit 1 (*N* = 16)	Visit 2 (*N* = 16)	*P*-value
eGFR (mL/min/1.73 m^2^)		89.5 (84.25; 103.5)	95 (79.25; 100.5)	.586
Blood leucocytes (10^3^/mm^3^)		6.44 (5.6; 7.4)	6.49 (5.45; 7.2)	.865
	NA	0	1	
CRP >5 mg/mL	N	13 (86.7)	11 (84.6)	1
	Y	2 (13.3)	2 (15.4)	
	NA	1	3	
U specific gravity		1015 (1015; 1020)	1015 (1010; 1015)	.188
	NA	3	5	
U pH		6.8 (6.3; 7)	7 (7; 7.4)	.022
	NA	1	3	
U cystine crystals	N	7 (43.7)	6 (42.9)	1
	Y	9 (56.2)	8 (57.1)	
	NA	0	2	
Stones^a^	N	3 (20)	2 (15.4)	1
	Y	12 (80)	11 (84.6)	
	NA	1	3	
U leucocytes >10/mm^3^	N	9 (56.2)	11 (68.7)	.617
	Y	7 (43.7)	5 (31.2)	
U red blood cells >10/mm^3^	N	6 (37.5)	12 (75)	.041
	Y	10 (62.5)	4 (25)	
U protein/creatinine ratio >30 mg/mmol	N	15 (93.7)	12 (100)	1
	Y	1 (6.2)	0 (0)	
	NA	0	4	

Biochemical parameters were described using median (interquartile range) for continuous variables and *n* (%) for qualitative variables.

^a^Presence of calculi in the renal cavities or in the urinary tract.

Visit 1 was the first visit before the onset of the alkalizing treatment, and Visit 2 was the second visit after 3 months of alkalizing treatment.

N: no; Y: yes; NA: missing data; U: urinary.

## DISCUSSION

We found a higher urinary abundance of 11 inflammatory proteins, constituting a new UIS, in Cys-patients without CKD, compared with HC. In patients with a very high/high baseline UIS, the alkalizing treatment was associated to a decrease in this UIS.

Among the 38 proteins upregulated in Cys-patients versus controls in our previous work [10], 11 proteins (6 neutrophil-derived proteins and 5 circulating inflammatory proteins) were also upregulated in Cys-patients without CKD compared with HC in the present study. Most of the remaining 27 proteins were also found elevated in Cys-patients in this study, but their increase was not significant, suggesting a smaller deregulation and inter-patients variability for these markers. This may be explained by the fact that patients in the present study had no CKD and consequently fewer deregulated proteins than in our previous study, showing that the UIS was more pronounced in Cys-patients with CKD [10]. As in our previous study [10], we confirm the higher abundance of the protein rBAT, which is a subunit of the dibasic amino acid transporter, in HC compared with Cys0-patients. This result strengthens the findings of our study. The abundance of rBAT protein was not significantly different between AA and BB Cys-patients, likely because both subunits have to be co-expressed to reach the plasma membrane [11–13].

Importantly, using this UIS, we could highlight two main clusters of Cys-patients without CKD and show that some of them had a very high/high UIS. It is worth underlining that Cys-patients with a very high/high UIS had a similar eGFR to those with a moderate/low UIS, showing that this UIS may indeed be present before eGFR alteration. These patients were also significantly younger when the first symptoms of cystinuria occurred, suggesting that they have a more severe form of the disease and highlighting the interest of studying UIS in pediatrics. Kovacevic *et al*. previously identified proteins associated with oxidative stress and inflammatory response in urine of two cystinuric children compared with healthy controls [14].

Surprisingly, we found a lower prevalence of urinary cystine crystals in patients with a very high/high UIS compared with those with a moderate/low UIS. Our hypothesis is that massive intra-tubular cystine crystals precipitation may lead to a reduction in urinary cystine crystals excretion but also to tubulo-interstitial inflammation with neutrophils recruitment, explaining the increased prevalence of aseptic leucocyturia as well as the intensity of the UIS. In line with this hypothesis, biopsies of the papillae of Cys-patients showed that many Bellini ducts were plugged with cystine crystals, and had injured or absent lining cells with a surrounding interstitium that was inflamed to fibrotic [15]. Cystine crystals can also induce interleukin-1 secretion, associated with inflammasome activation [16], and renal interstitial inflammation is found in animal models of cystinuria [17–19]. The intensity of the UIS in non-treated Cys-patients was not associated with CRP, blood leukocytes, the presence of calculi, recent urinary tract infections, urological interventions or spontaneous calculi expulsions, suggesting that this UIS may be linked to an intrarenal inflammatory process, as suggested by the higher prevalence of leukocyturia in patients with a strong UIS, rather than to systemic or urological inflammatory processes. Interestingly, whereas urinary cystine crystals are associated with cystine stones recurrence [20], UIS intensity is associated with lower prevalence of urine cystine crystals. UIS intensity could indeed be a specific marker of renal inflammation and of the evolution toward CKD in cystinuria.

Inflammation in the urine proteome of 10 Cys-patients (versus HC) was previously reported, with, as in our study, an upregulation of *A2M, C3, ELANE* and *DEFA1* [21], but the other proteins of our UIS were not mentioned [21]. However, in this study, some patients were treated and eGFR was sometimes lacking [21], which could have affected the urine proteome profile [10]. Urinary proteomics in other rare kidney stone diseases [22] has identified specific proteins, for example, to discriminate medullary sponge kidney disease from idiopathic calcium nephrolithiasis or autosomal dominant polycystic kidney disease [23–25] or type 1 primary hyperoxaluria from idiopathic hypercalciuria [26]. Interestingly, although some similar urinary proteins are found in rare kidney stone disease and in idiopathic calcium nephrolithiasis, differences are reported. In our study, which focused only on the UIS, we found that the UIS was not specific to cystinuria, although it was more intense in some Cys-patients, since it could also be found in patients with common calcium nephrolithiasis.

One of the key messages of our study is that conventional alkalizing treatment induces a decrease in the intensity of the UIS in Cys-patients who have a very high/high baseline UIS, with a significant decrease in the intensity of 5/6 urinary neutrophil-derived proteins and, to a lesser extent, a decrease in urinary inflammatory circulating proteins constituting this UIS. It is possible that inflammatory circulating proteins could decrease significantly after a longer period of alkalizing treatment. It would thus be possible to consider these five neutrophil-derived proteins (namely Neutrophil elastase, Cathepsin G, Matrix metalloproteinase-9, Proteinase 3 and Coronin-1A) as a tool to monitor the renal “parenchymal” effect of the alkalizing treatment. Interestingly, this result points out the potential role of neutrophils in the pathogenicity of the renal involvement due to cystinuria and its potential reversibility upon treatment. A study of the UIS and of the effect of alkalization in a mouse model for cystinuria [17, 27] could help to understand the pathways linking alkalization and UIS, in order to assess whether it is an effect on crystallization or on another mechanism, such as ammoniogenesis for example [28].

Our study has weaknesses. First, Cys0-patients were significantly younger than Lith-patients and HC, reflecting the real demography of patients in our centers. Second, the baseline _U_pH was higher in Cys-patients although they did not receive any alkalizing agent, suggesting that they already had a more alkalizing diet. Third, although it was significantly increased, _U_pH remained below the recommended targets of 7.5–8 [4, 5] in most treated Cys-patients, suggesting that we may have observed a more pronounced effect of the alkalizing treatment if those targets had been reached. The limited number of patients included in our study decreases the power of the statistical analyses and limits the subgroups analyses. Importantly, choosing Cys-patients that were not taking alkalizing treatment in the last 3 months may have biased the results as such patients may have a less severe disease. However, the Cys-patients included in our study were either patients lost to follow-up or newly diagnosed Cys-patients, and most of them had kidney stones.

Our study also has several strengths, since it included the largest number of Cys-patients for this purpose with a control group of other types of nephrolithiasis and an estimate of GFR for all patients and controls. Finally, for the first time, the effect of urine alkalization on urine proteomics has been assessed in Cys-patients.

In conclusion, we report different degrees of UIS involving 11 proteins overexpressed in cystinuric patients without CKD compared with HC. In Cys-patients with a high UIS, the alkalizing treatment is associated with a significant decrease in urinary neutrophil-derived proteins. Whether this UIS could be used as a marker to predict the clinical course of cystinuria and the potential evolution towards CKD remains to be determined through longitudinal studies.

## Supplementary Material

sfae040_Supplemental_File

## Data Availability

Data from the present study will be made available upon reasonable request. The mass spectrometry proteomics data have been deposited to the ProteomeXchange Consortium via the PRIDE partner repository with the dataset identifier PXD035631. The correspondence table between the patient groups and the data loaded in the public repository is available in Supplementary data, Table S5.
