# Supplementary material for: Effect of urine alkalization on urinary inflammatory markers in cystinuric patients
Source: Clin Kidney J. 2024 Feb 22;17(3):sfae040. doi: 10.1093/ckj/sfae040 (PMC10953617; doi:10.1093/ckj/sfae040)
Supplement: sfae040_Supplemental_File [file sfae040_supplemental_file.zip › 1173 suppl mat.docx]

**Supplementary file**

***Table of contents***

**Supplemental material**

**Nano LC-MS/MS protein identification and quantification**

**Statistical Analyses**

**Supplementary figures**

**Supplementary Figure S1. Comparison of neutrophil-derived proteins (panel A) and inflammatory circulating proteins (panel B) intensity profile between the Cys0, Lith and HC groups**

**Supplementary Figure S2. Comparison of neutrophil-derived proteins (panel A) and inflammatory circulating proteins (panel B) intensity profile between AA and BB cystinuric patients**

**Supplementary Figure S3. Comparison of neutrophil-derived proteins (panel A) and inflammatory circulating proteins (panel B) intensity profile before and after a 3-month alkalizing treatment for the 5 cystinuric patients having a very high/high urinary baseline inflammatory signature**

**Supplementary tables**

**Supplementary Table S1: Baseline characteristics of patients with cystinuria (Cys0-patients) and of patients with other types of nephrolithiasis (Lith-patients)**

**Supplementary Table S2: Genes and proteins names of the 11 proteins of the urinary inflammatory signature**

**Supplementary Table S3: distribution of patients of each group according to the intensity of the baseline urinary inflammatory signature.**

**Supplementary Table S4: Baseline characteristics of patients with cystinuria undergoing only the first visit (Cys0 without V2) or the two visits. before and after the 3-month alkalizing treatment (Cys1).**

**Supplementary Table S5: Correspondence table between the patient groups and the data loaded in the public repository**

**Supplementary references**

**Supplemental material**

**Nano LC-MS/MS protein identification and quantification**

Prior to digestion, protein precipitation was carried out on 1 mL of urine sample by addition of 100 µL of TCA 100% and stored overnight at -20°C. After centrifugation, pellets were then washed thrice with cold acetone at 4°C. Residue of acetone was evaporated at room temperature.

S-TrapTM micro spin column (Protifi, Hutington, USA) digestion was performed on protein pellets according to manufacturer’s protocol. Briefly, 5% SDS was added to the samples which were then boiled 3 min at 95°C. Proteins were reduced with the addition of TCEP to a final concentration of 20 mM and alkylated with the addition of chloroacetamide to a final concentration of 50 mM. Aqueous phosphoric acid was added to a final concentration of 1.2%. Colloidal protein particulate was formed with the addition of 6 times the sample volume of S-Trap binding buffer (90% aqueous methanol, 100 mM TEAB, pH 7.1). The mixtures were put on the S-Trap micro 1.7mL columns and centrifuged at 4,000 g for 30 seconds. The columns were washed four times with 150 µL S-Trap binding buffer and centrifuged at 4,000 g for 30 seconds with 180 degrees rotation of the columns between washes. Samples were digested with 4 µg of trypsin (Promega) at 47°C for 2 hrs.

Dried ~~s~~Samples were resuspended in 50 µL of 10% ACN, 0.1% TFA in HPLC-grade water. Each sample was injected three times. For each run, 5 µL was injected in a nanoRSLC-Q Exactive PLUS (RSLC Ultimate 3000) (Thermo Scientific, Waltham MA, USA). Peptides were loaded onto a µ-precolumn (Acclaim PepMap 100 C18, cartridge, 300 µm i.d.×5 mm, 5 µm) (Thermo Scientific), and were separated on a 50 cm reversed-phase liquid chromatographic column (0.075 mm ID, Acclaim PepMap 100, C18, 2 µm) (Thermo Scientific). Chromatography solvents were (A) 0.1% formic acid in water, and (B) 80% acetonitrile, 0.08% formic acid. Peptides were eluted from the column with the following gradient 5% to 40% B (120 minutes), 40% to 80% (1 minutes). At 121 minutes, the gradient stayed at 80% for 5 minutes and, at 126 minutes, it returned to 5% to re-equilibrate the column for 20 minutes before the next injection. One blank was run between each replicate to prevent sample carryover. Peptides eluting from the column were analyzed by data dependent MS/MS, using top-10 acquisition method. Peptides were fragmented using higher-energy collisional dissociation (HCD). Briefly, the instrument settings were as follows: resolution was set to 70,000 for MS scans and 17,500 for the data dependent MS/MS scans in order to increase speed. The MS AGC target was set to 3.106 counts with maximum injection time set to 200 ms, while MS/MS AGC target was set to 1.105 with maximum injection time set to 120 ms. The MS scan range was from 400 to 2000 m/z. Dynamic exclusion was set to 30 seconds duration.

The MS files were processed simultaneously with the MaxQuant software version 1.6.6.0 and searched with Andromeda search engine against the UniProtKB/Swiss-Prot Homo sapiens database (release 15-04-2019, 20415 entries). To search parent mass and fragment ions, we set a mass deviation of 3 ppm and 20 ppm respectively. The minimum peptide length was set to 7 amino acids and strict specificity for trypsin cleavage was required, allowing up to two missed cleavage sites. Carbamidomethylation (Cys) was set as fixed modification, whereas oxidation (Met) and N-term acetylation were set as variable modifications. The false discovery rates (FDR) at the protein and peptide level were set to 1%. Scores were calculated in MaxQuant as described previously^1^. The reverse and common contaminants hits were removed from MaxQuant output. Proteins were quantified according to the MaxQuant label-free algorithm using LFQ intensities; protein quantification was obtained using at least 2 peptides per protein. Match between runs was allowed.

**Statistical Analyses**

Statistical and bioinformatic analysis, including heatmaps, profile plots and clustering, were performed with Perseus software (version 1.6.15.0) freely available at [www.perseus-framework.org](http://www.perseus-framework.org) ^2^. For statistical comparison, we set different groups: 7 HC, 12 Lith-patients, 21 cystinuric non treated patients (Cys0-patients), and among them, 16 cystinuric patients that had correctly completed the 3-month alkalizing treatment (Cys1-patients). Raw intensity values underwent a Log2 transformation and normalization using the "width adjustment" option in Perseus. This involves calculating quartiles (q1, q2, q3) from the overall value distribution. The median is subtracted from each value to center the distribution, followed by asymmetric division based on positivity or negativity. Positive values are divided by q3-q2, and negative values by q2-q1. A constant is then added to all values to handle positive numbers. We filtered the data to keep only proteins with at least 70% of valid values in total. Next, the data were imputed to fill missing data points by creating a Gaussian distribution of random numbers with a standard deviation of 30% relative to the standard deviation of the measured values and 1.8 standard deviation downshift of the mean to simulate the distribution of low signal values. We performed different t-test with FDR threshold valued indicated in each figure. Hierarchical clustering of proteins that survived the test was performed in Perseus on logarithmized label-free quantification intensities after z-score normalization of the data, using Euclidean distances.

Statistical analysis regarding the expression of inflammatory proteins were performed using R (version 4.1.2) and RStudio (RStudio 2021.09.1+372 "Ghost Orchid" Release). For this purpose, paired t-tests were performed for each protein (corresponding gene name: ELANE, CTSG, MMP9, PRTN3, CORO1A, DEFA3/DEFA1, A2M, C3, FGB, FGG, ITH2) using 2 groups composed of 5 identical patients before and after taking treatment. Parametric paired t-test was performed using ggstatsplot^3^ R package (version 0.9.3) with the ggwithinstats function with a confidence level of 0.95 and subsequent p-value and Fold Change (FC) were indicated in each boxplot. Urinary inflammatory profile on the 21 Cys0-patients, 12 Lith-patients and 7 HC was also investigated using parametric one-way ANOVA (3 groups) with the ggbetweenstats function. A confidence level of 0.95 was used and subsequent *P*-values were corrected using Benjamini Hochberg false discovery rate method and were indicated in each boxplot.

**Supplementary figures**


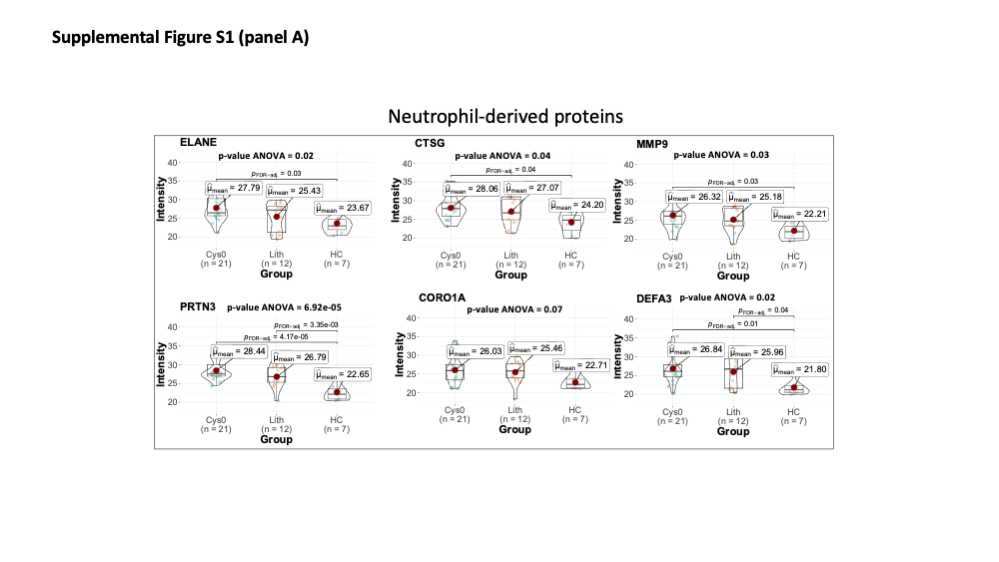


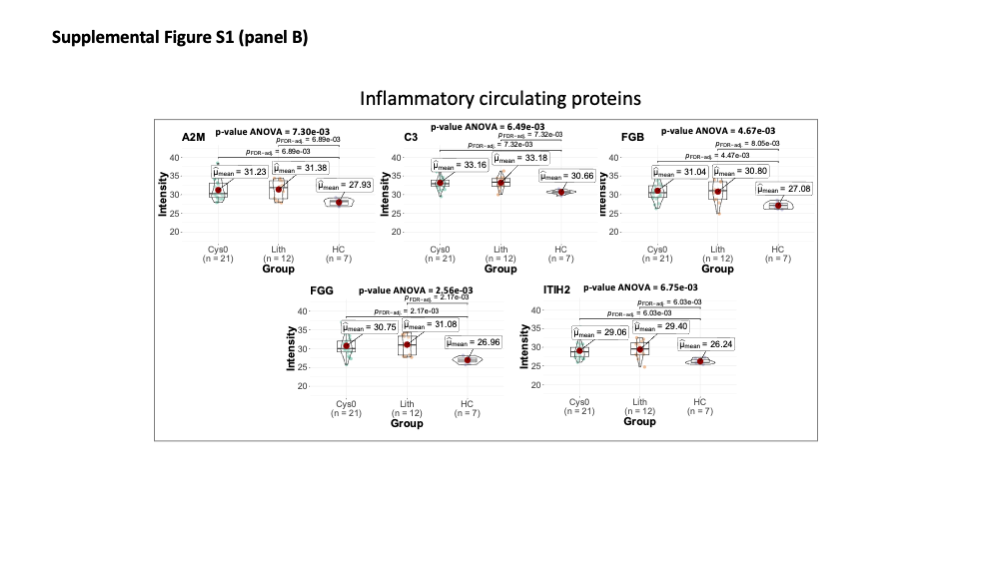


**Supplementary Figure S1. Comparison of neutrophil-derived proteins (panel A) and inflammatory circulating proteins (panel B) intensity profile between the Cys0, Lith and HC groups.** Boxplot representation of parametric one-way ANOVA performed on the following groups: Cys0: Non-treated cystinuric patients (n=21), Lith: patients with other types of nephrolithiasis (n=12), HC: healthy controls (n=7). The global ANOVA *P*-value is represented above each boxplot and only pairwise significant comparison are represented with their respective p_FDR-adj_ (adjusted *P*-value after Benjamini Hochberg multiple testing correction). For the name of the protein corresponding to the gene name, refer to sup. table 2.


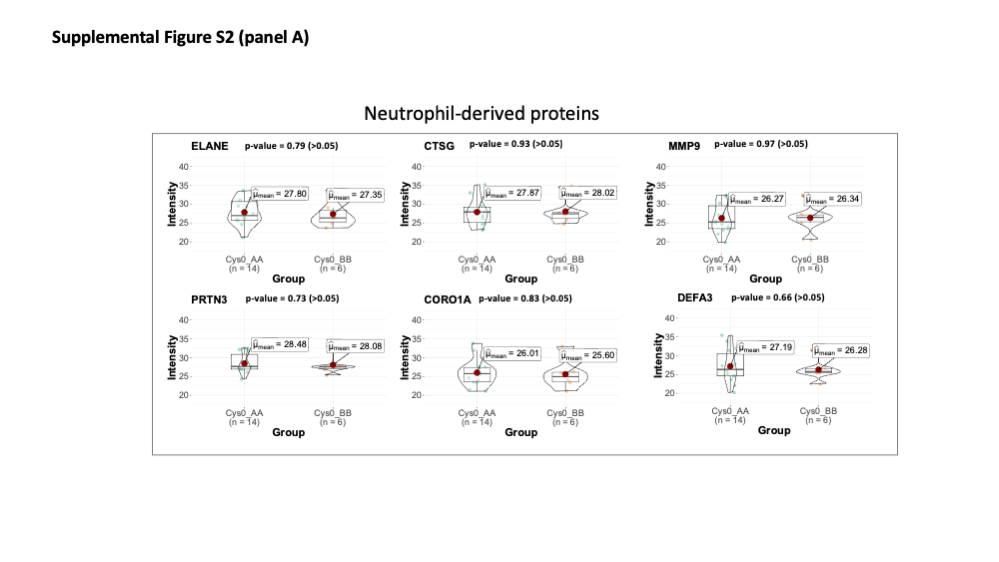


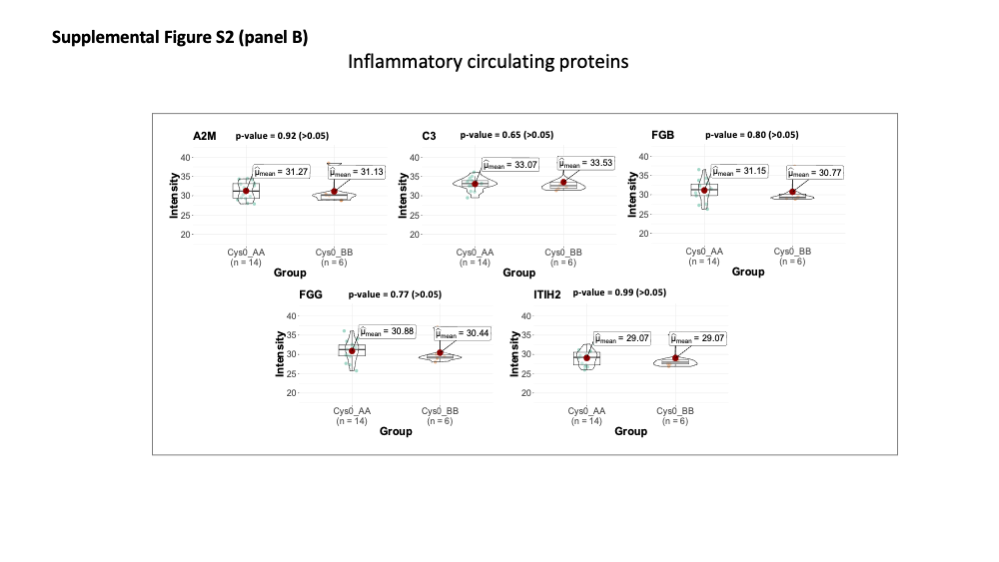


**Supplementary Figure S2. Comparison of neutrophil-derived proteins (panel A) and inflammatory circulating proteins (panel B) intensity profile between AA and BB cystinuric patients.** Boxplot representation of parametric Student’s t-test performed on the following groups: Cys0_AA: Non-treated cystinuric patients having AA genotype (n=14), Cys0_BB: Non-treated cystinuric patients having BB genotype (n=6). The global t-test *P*-value (p) is represented above each boxplot and for the name of the protein corresponding to the gene name, refer to sup. table 2.


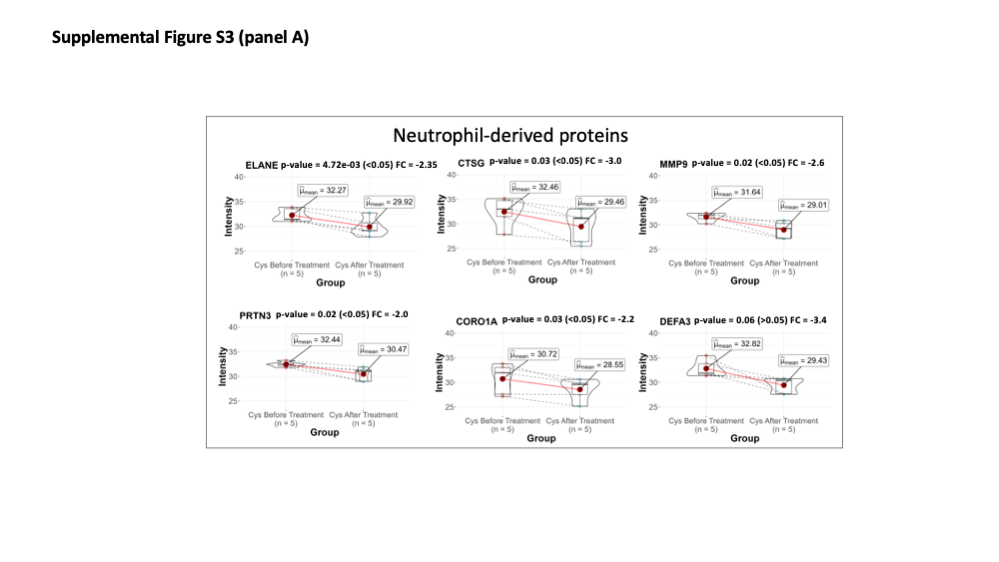


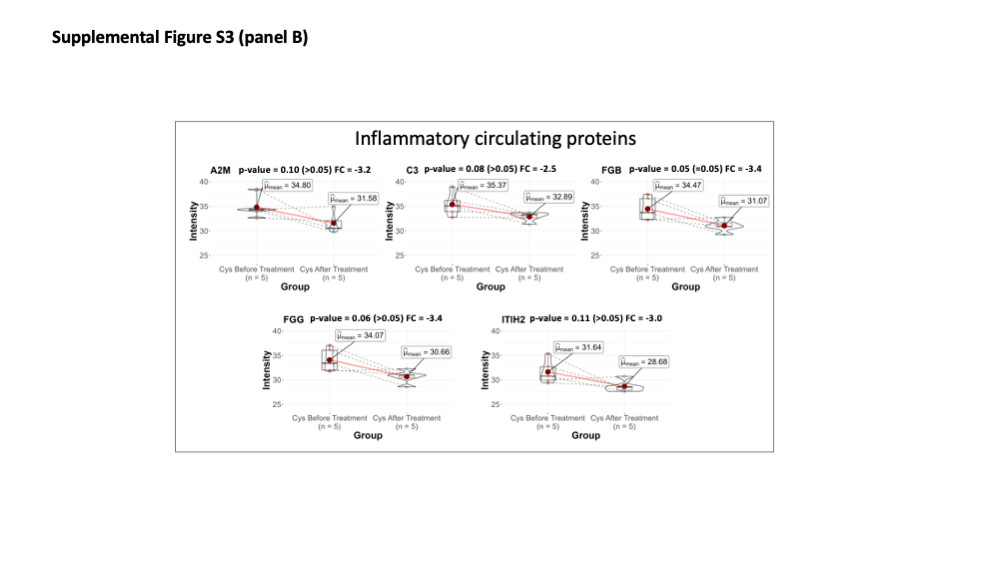


**Supplementary Figure S3. Comparison of neutrophil-derived proteins (panel A) and inflammatory circulating proteins (panel B) intensity profile before and after a 3-month alkalizing treatment for the 5 cystinuric patients having a very high/high urinary baseline inflammatory signature.** Boxplots represent parametric paired t-test performed on neutrophil-derived proteins and inflammatory circulating proteins between cystinuric patients before and after treatment.

**Supplementary tables**

|  |  | **Cys0-patients** | **Lith-patients** | ***P*-value** |
| --- | --- | --- | --- | --- |
|  |  | (N=21) | (N=12) |  |
| **Age (years)** |  | 29.92 [24.6; 42.8] | 46.25 [39.5; 54.2] | 0.03 |
| **Sex** | W | 9 (42.9) | 4 (33.3) | 0.719 |
|  | M | 12 (57.1) | 8 (66.7) |  |
| **BMI (kg/m^2^)** |  | 24.4 [24.1; 29] | 26.35 [22.7; 28.9] | 0.955 |
| **Age at first symptoms (years)** |  | 17 [13; 20] | 31.45 [24.1; 35.6] | 0.006 |
| **Diabetes mellitus** | N | 21 (100) | 8 (66.7) | 0.012 |
|  | Y | 0 (0) | 4 (33.3) |  |
| **High blood pressure** | N | 20 (95.2) | 9 (75) | 0.125 |
|  | Y | 1 (4.8) | 3 (25) |  |
| **Other kidney disease** | N | 20 (95.2) | 10 (83.3) | 0.538 |
|  | Y | 1 (4.8) | 2 (16.7) |  |
| **eGFR (mL/min/1.73m^2^)** |  | 95 [88; 111] | 106 [97.5; 117.7] | 0.134 |
| **Blood leucocytes (10^3^/mm^3^)** |  | 5.95 [5.2; 7.6] | 6.3 [5.1; 7.2] | 0.722 |
| **CRP >5 mg/L** | N | 17 (85) | 11 (91.7) | 1 |
|  | Y | 3 (15) | 1 (8.3) |  |
|  | NA | 1 | 0 |  |
| **Renin angiotensin inhibitors treatment** | N | 20 (95.2) | 9 (75) | 0.125 |
|  | Y | 1 (4.8%) | 3 (25) |  |
| **U specific gravity** |  | 1015 [1015; 1020] | 1012 [1006.5; 1020] | 0.394 |
|  | NA | 3 | 1 |  |
| **U pH** |  | 6.65 [6; 7] | 5.9 [5.4; 6] | 0.002 |
|  | NA | 1 | 1 |  |
| **U crystals** | N | 5 (23.8) | 7 (58.3) | 0.047 |
|  | Y | 16 (76.2) | 5 (41.7) |  |
| **Stones**** | N | 5 (25) | 3 (27.3) | 1 |
|  | Y | 15 (75) | 8 (72.7) |  |
|  | NA | 1 | 1 |  |
| **Urological intervention*** | N | 16 (76.2) | 8 (66.7) | 0.69 |
|  | Y | 5 (23.8) | 4 (33.3) |  |
| **U leucocytes ≥10/mm^3^** | N | 13 (61.9) | 7 (58.3) | 0.84 |
|  | Y | 8 (38.1) | 5 (41.7) |  |
| **U red blood cells ≥10/mm^3^** | N | 10 (47.6) | 7 (58.3) | 0.554 |
|  | Y | 11 (52.4) | 5 (41.7) |  |
| **U protein/creatinine ratio>30 mg/mmol** | N | 20 (95.2) | 11 (91.7) | 1 |
|  | Y | 1 (4.8) | 1 (8.3) |  |

**Supplementary Table S1: Baseline characteristics of patients with cystinuria (Cys0-patients) and of patients with other types of nephrolithiasis (Lith-patients).** M: men. W: women. N: no. Y: yes. NA: missing data. U: urinary, BMI: body mass index, CRP: circulating C reactive protein, eGFR: estimated glomerular filtration rate using the CKD-EPI formula. Baseline characteristics were described using median [interquartile range] for continuous variables and n (%) for qualitative variables. *: in the 6 months prior to V1. ** presence of calculi in the renal cavities or in the urinary tract

| **Gene symbol** | **Protein names** |
| --- | --- |
| ***Inflammatory circulating proteins*** | |
| *A2M* | Alpha-2-macroglobulin |
| *C3* | Complement C3 |
| *FGB* | Fibrinogen beta chain |
| *FGG* | Fibrinogen gamma chain |
| *ITIH2* | Inter-alpha-trypsin inhibitor heavy chain H2 |
| ***Neutrophil-derived proteins*** | |
| *ELANE* | Neutrophil elastase |
| *CTSG* | Cathepsin G |
| *MMP9* | Matrix metalloproteinase-9 |
| *PRTN3* | Proteinase 3 |
| *CORO1A* | Coronin-1A |
| *DEFA3; DEFA1* | Neutrophil defensin 3; Neutrophil defensin 1 |

**Supplementary Table S2: Genes and proteins names of the 11 proteins of the urinary inflammatory signature**

| **baseline urinary inflammatory signature** | **Cys0** | **Lith** | **HC** | ***P*-value** |
| --- | --- | --- | --- | --- |
|  | (N=21) | (N=12) | (N=7) | 0.004 |
| **1 - strong** | 5 (23.8) | 2 (16.7) | 0 (0) |  |
| **2 - moderate** | 7 (33.3) | 4 (33.3) | 0 (0) |  |
| **3 - low** | 8 (38.1) | 5 (41.7) | 1 (14.3) |  |
| **4 - no** | 1 (4.8) | 1 (8.3) | 6 (85.7) |  |

**Supplementary Table S3: distribution of patients of each group according to the intensity of the baseline urinary inflammatory signature.** Cys0: non-treated patients with cystinuria, Lith: patients with other types of nephrolithiasis, HC: healthy controls. Proportions expressed as n (%).

|  |  | **Cys0 without V2** | **Cys1** | ***P-*value** |
| --- | --- | --- | --- | --- |
|  |  | (N=5) | (N=16) |  |
| **Age (years)** |  | 31.1 [27.4; 38.9] | 29.4 [23.7; 43.4] | 0.719 |
| **Sex** | W | 2 (40%) | 7 (43.7%) | 1 |
|  | M | 3 (60%) | 9 (56.2%) |  |
| **BMI (kg/m^2^)** |  | 24.2 [24.2; 29] | 24.45 [23.8; 28.8] | 0.869 |
| **Age at first symptoms (years)** |  | 16 [16; 38] | 17 [9.7; 19.2] | 0.407 |
| **Diabetes mellitus** | N | 5 (100%) | 16 (100%) | --- |
| **High blood pressure** | N | 5 (100%) | 15 (93.7%) | 1 |
|  | Y | 0 (0%) | 1 (6.2%) |  |
| **Other kidney disease** | N | 5 (100%) | 15 (93.7%) | 1 |
|  | Y | 0 (0%) | 1 (6.2%) |  |
| **Genotype** | AA | 1 | 13 | 0.061 |
|  | BB | 3 | 3 |  |
|  | NA | 1 |  |  |
| **eGFR (mL/min/1.73m^2^)** |  | 113 [95; 122] | 89.5 [84.2; 103.5] | 0.043 |
| **Blood leucocytes (10^3^/mm^3^)** |  | 5.2 [5.2; 8] | 6.44 [5.6; 7.4] | 0.773 |
| **CRP >5 mg/L** | N | 4 (80%) | 13 (86.7%) | 1 |
|  | Y | 1 (20%) | 2 (13.3%) |  |
|  | NA | 0 | 1 |  |
| **Renin angiotensin inhibitors treatment** | N | 5 (100%) | 15 (93.7%) | 1 |
|  | Y | 0 (0%) | 1 (6.2%) |  |
| **Anti inflammatory treatment*** | N | 4 (100%) | 14 (93.3%) | 1 |
|  | Y | 0 (0%) | 1 (6.7%) |  |
|  | NA | 1 | 1 |  |
| **Urinary tract infection*** | N | 4 (80%) | 14 (87.5%) | 1 |
|  | Y | 1 (20%) | 2 (12.5%) |  |
| **Spontaneous expulsion of stones*** | N | 4 (80%) | 8 (57.1%) | 0.603 |
|  | Y | 1 (20%) | 6 (42.9%) |  |
|  | NA | 0 | 2 |  |
| **Urological intervention*** | N | 4 (80%) | 12 (75%) | 1 |
|  | Y | 1 (20%) | 4 (25%) |  |
| **U specific gravity** |  | 1020 [1015; 1020] | 1015 [1015; 1020] | 0.604 |
|  | NA | 0 | 3 |  |
| **U pH** |  | 5.9 [5.9; 7.4] | 6.8 [6.3; 7] | 0.69 |
|  | NA | 0 | 1 |  |
| **U cystine crystals** | N | 1 (20%) | 7 (43.7%) | 0.606 |
|  | Y | 4 (80%) | 9 (56.2%) |  |
| **Other type of U crystals** | N | 1 (25%) | 11 (73.3%) | 0.117 |
|  | Y | 3 (75%) | 4 (26.7%) |  |
|  | NA | 1 | 1 |  |
| **Stone**** | N | 2 (40%) | 3 (20%) | 0.56 |
|  | Y | 3 (60%) | 12 (80%) |  |
|  | NA | 0 | 1 |  |
| **U leucocytes >10/mm^3^** | N | 4 (80%) | 9 (56.2%) | 0.606 |
|  | Y | 1 (20%) | 7 (43.7%) |  |
| **U red blood cells >10/mm^3^** | N | 4 (80%) | 6 (37.5%) | 0.149 |
|  | Y | 1 (20%) | 10 (62.5%) |  |
| **U protein/creatinine ratio>30 mg/mmol** | N | 5 (100%) | 15 (93.7%) | 1 |
|  | Y | 0 (0%) | 1 (6.2%) |  |

**Supplementary Table S4: Baseline characteristics of patients with cystinuria undergoing only the first visit (Cys0 without V2) or the two visits. before and after the 3-month alkalizing treatment (Cys1).** M: men. W: women. N: no. Y: yes. NA: missing data, U: urinary, BMI: body mass index, CRP: circulating C reactive protein, eGFR: estimated glomerular filtration rate using the CKD-EPI formula. Baseline characteristics were described using median [interquartile range] for continuous variables and n (%) for qualitative variables. * in the 6 months prior to V1. ** presence of calculi in the renal cavities or in the urinary tract

**Supplementary references**

1. Cox J, Mann M. MaxQuant enables high peptide identification rates, individualized p.p.b.-range mass accuracies and proteome-wide protein quantification. *Nat Biotechnol*. Dec 2008;26(12):1367-72.

2. Tyanova S, Temu T, Sinitcyn P, et al. The Perseus computational platform for comprehensive analysis of (prote)omics data. *Nat Methods*. Sep 2016;13(9):731-40.

3. Patil I. Visualizations with statistical details: The ‘ggstatsplot’

approach. *Journal of Open Source Software*. 2021;6(61):3167.
