# Supplementary figures and images for: Effect of urine alkalization on urinary inflammatory markers in cystinuric patients
Source: Clin Kidney J. 2024 Feb 22;17(3):sfae040. doi: 10.1093/ckj/sfae040 (PMC10953617; doi:10.1093/ckj/sfae040)

Supplemental Figure S1 (panel A)

Neutrophil-derived proteins

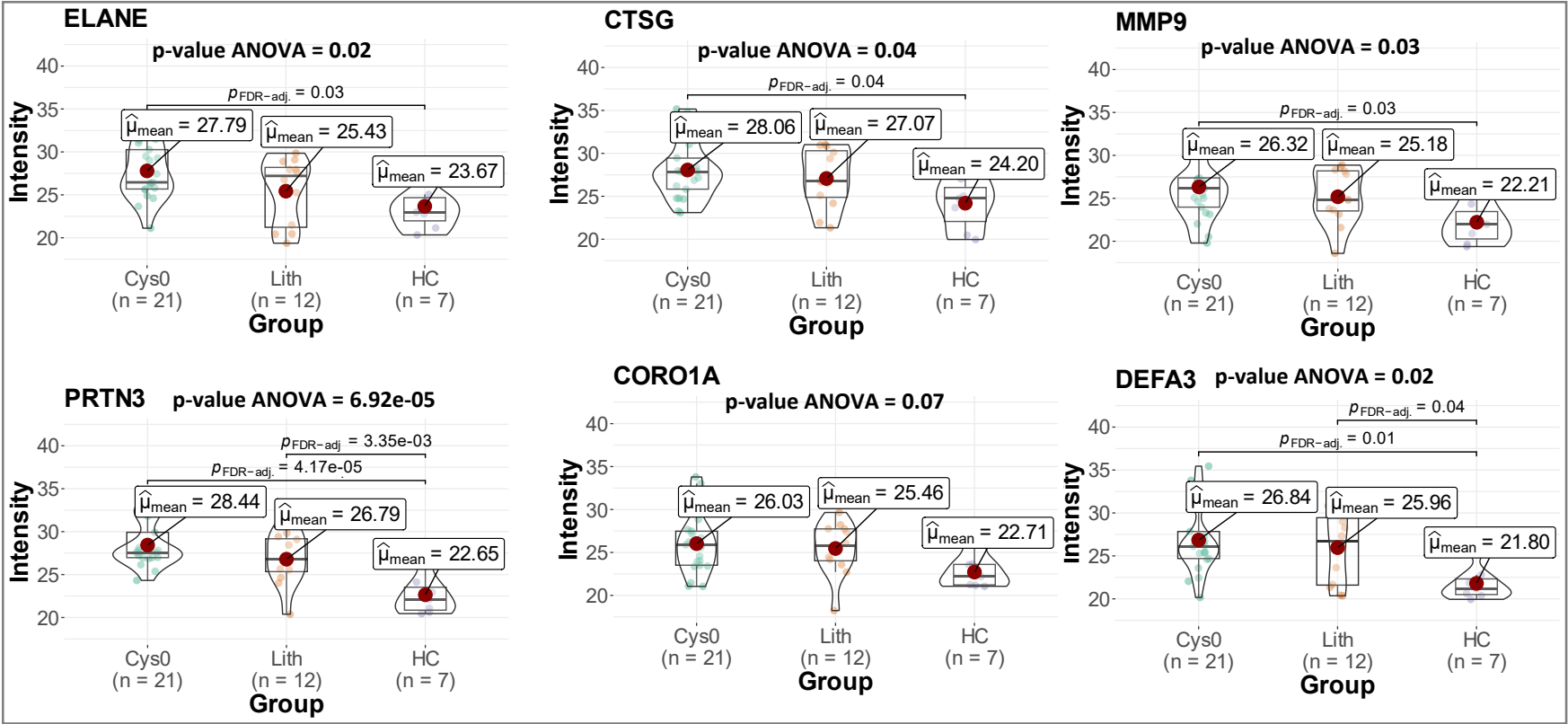

Supplement: sfae040_Supplemental_File [file sfae040_supplemental_file.zip › MaCO_Fig_S1_panelA.pdf]

Supplemental Figure S1 (panel B)

Inflammatory circulating proteins

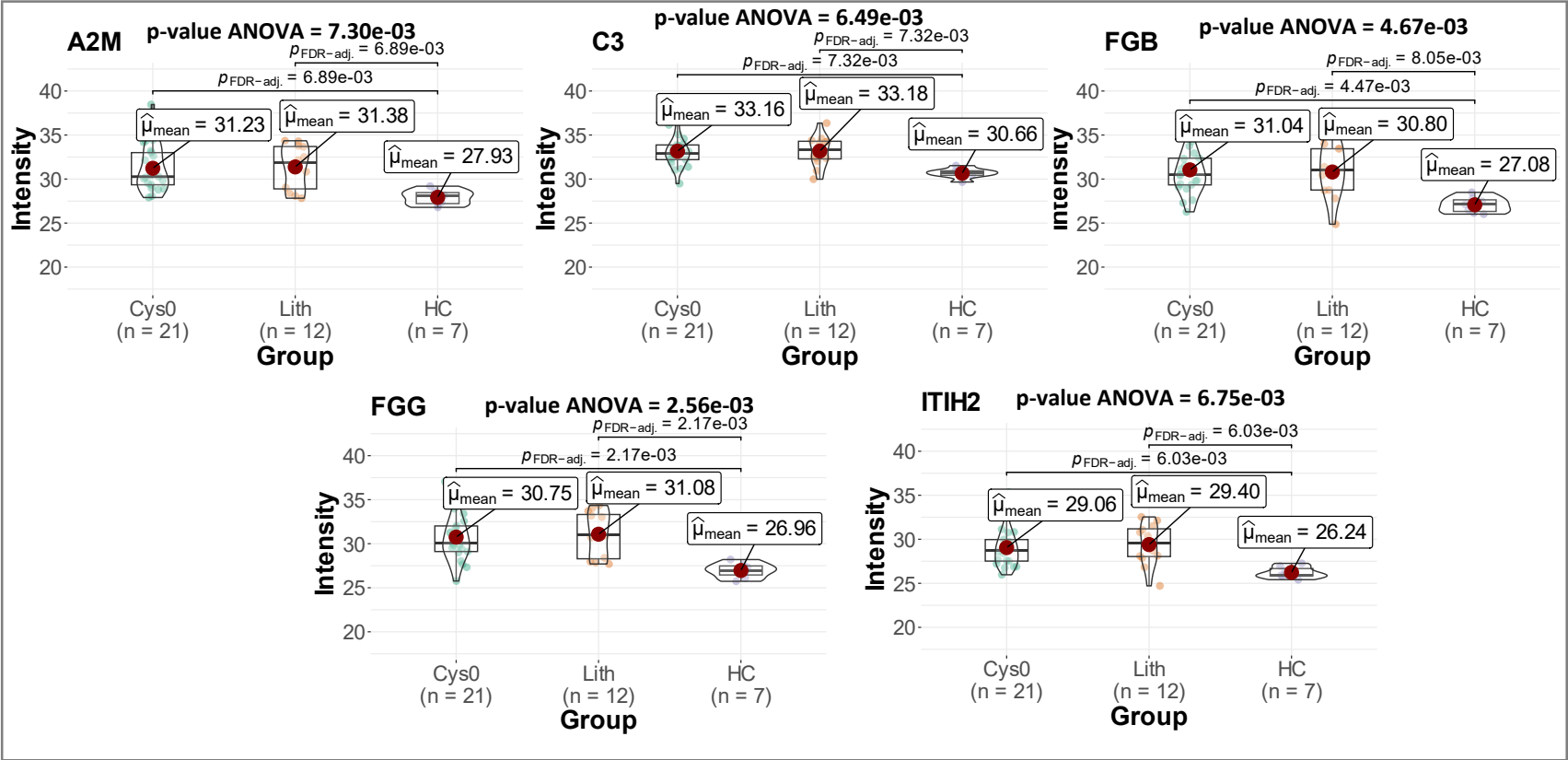

Supplement: sfae040_Supplemental_File [file sfae040_supplemental_file.zip › MaCO_Fig_S1_panelB.pdf]

Supplemental Figure S2 (panel A)

Neutrophil-derived proteins

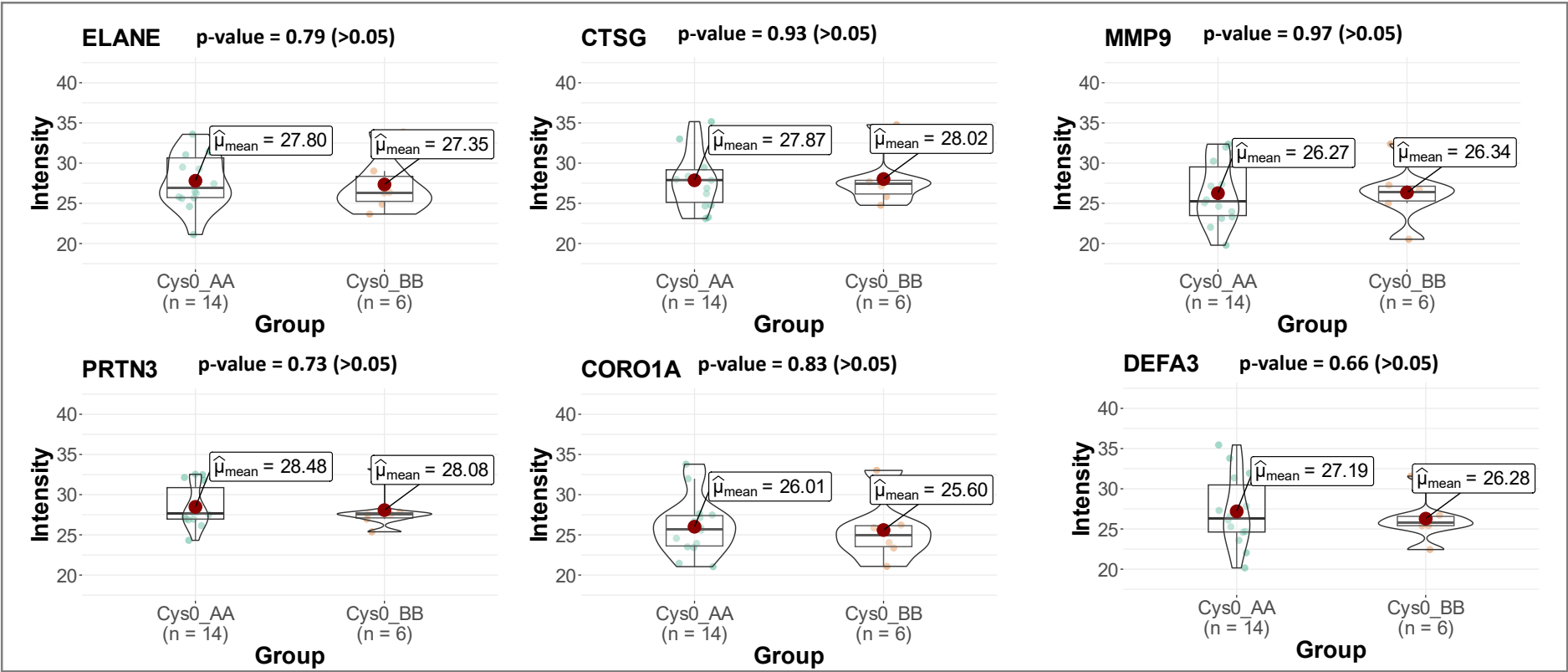

Supplement: sfae040_Supplemental_File [file sfae040_supplemental_file.zip › MaCO_Fig_S2_panelA.pdf]

Supplemental Figure S2 (panel B)

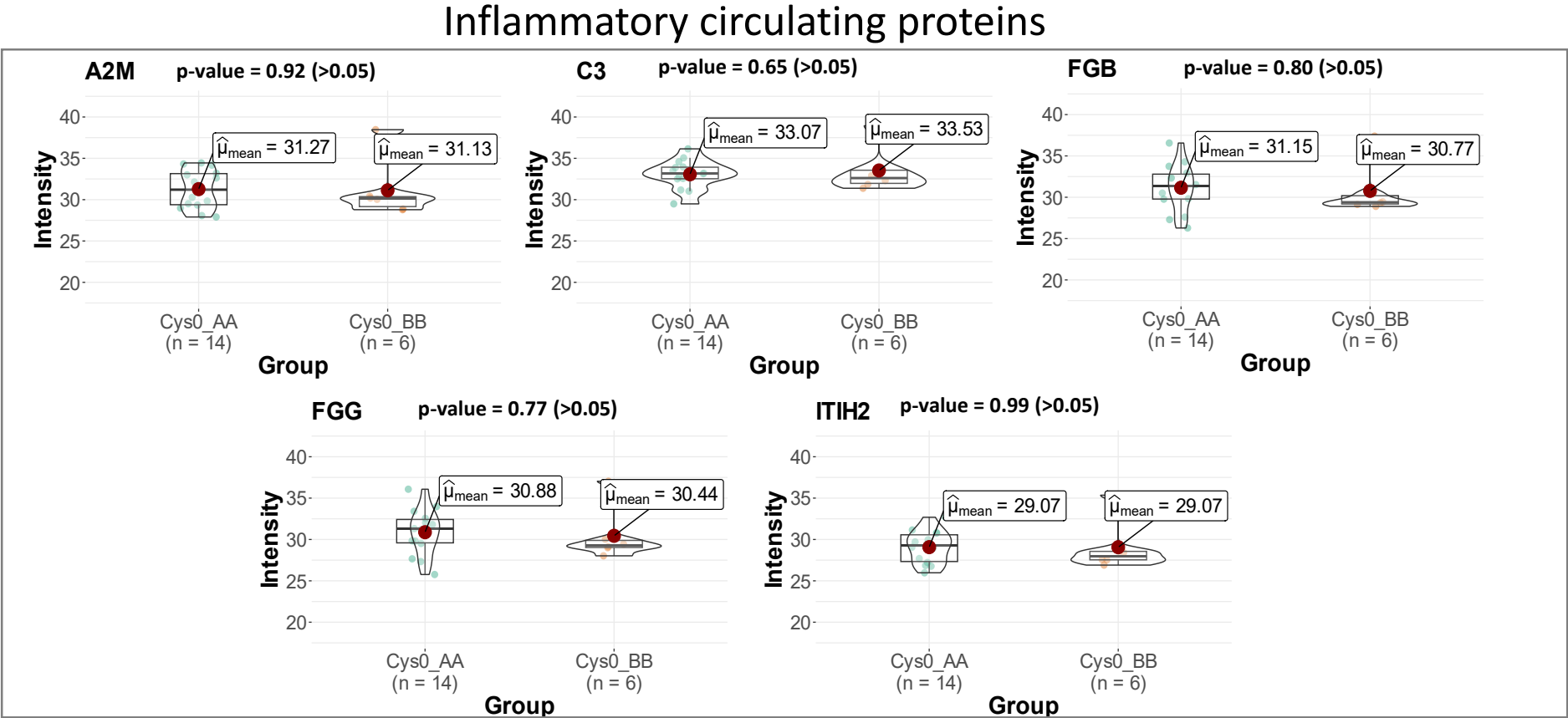

Supplement: sfae040_Supplemental_File [file sfae040_supplemental_file.zip › MaCO_Fig_S2_panelB.pdf]

Supplemental Figure S3 (panel A)

Neutrophil-derived proteins

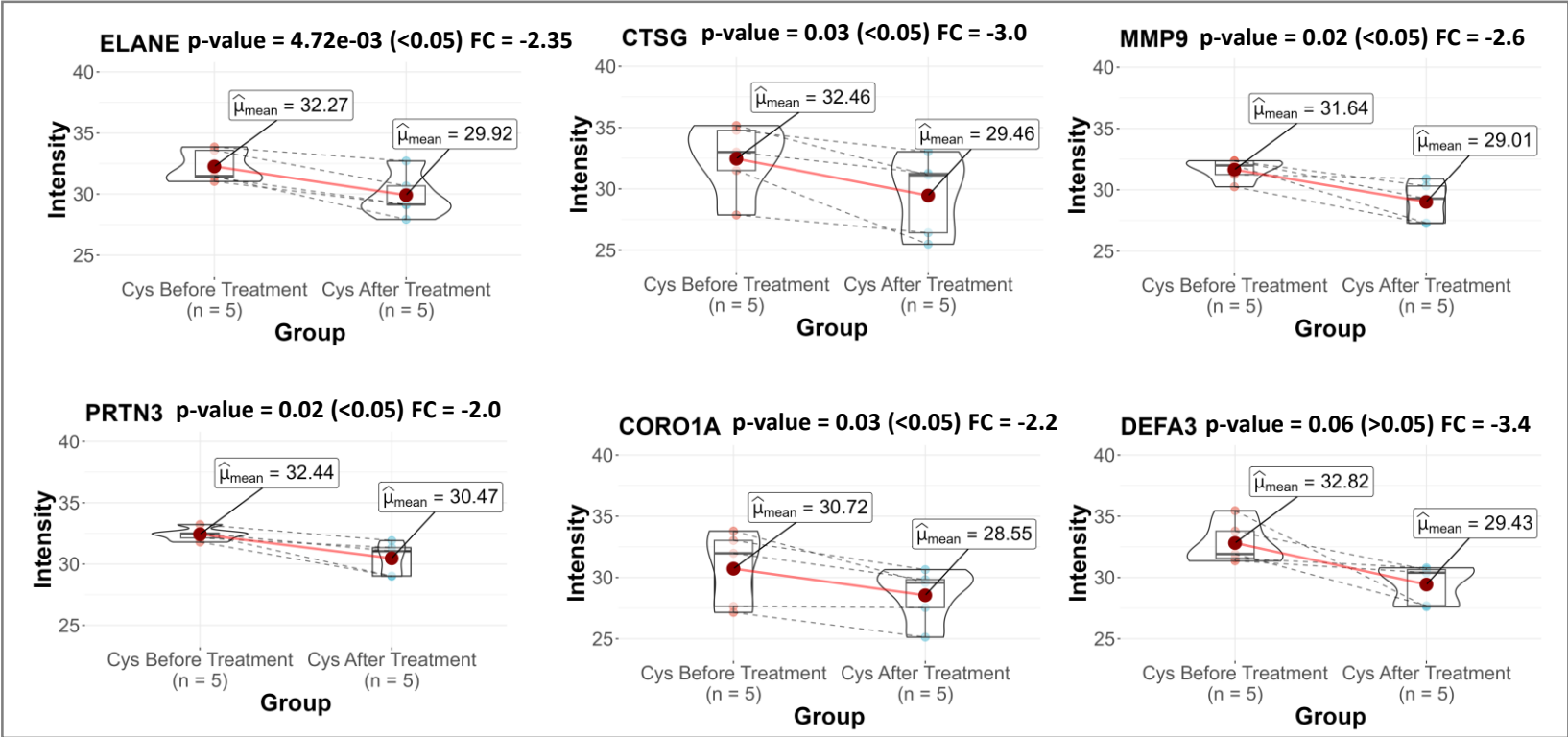

Supplement: sfae040_Supplemental_File [file sfae040_supplemental_file.zip › MaCO_Fig_S3_panelA.pdf]

Supplemental Figure S3 (panel B)

Inflammatory circulating proteins

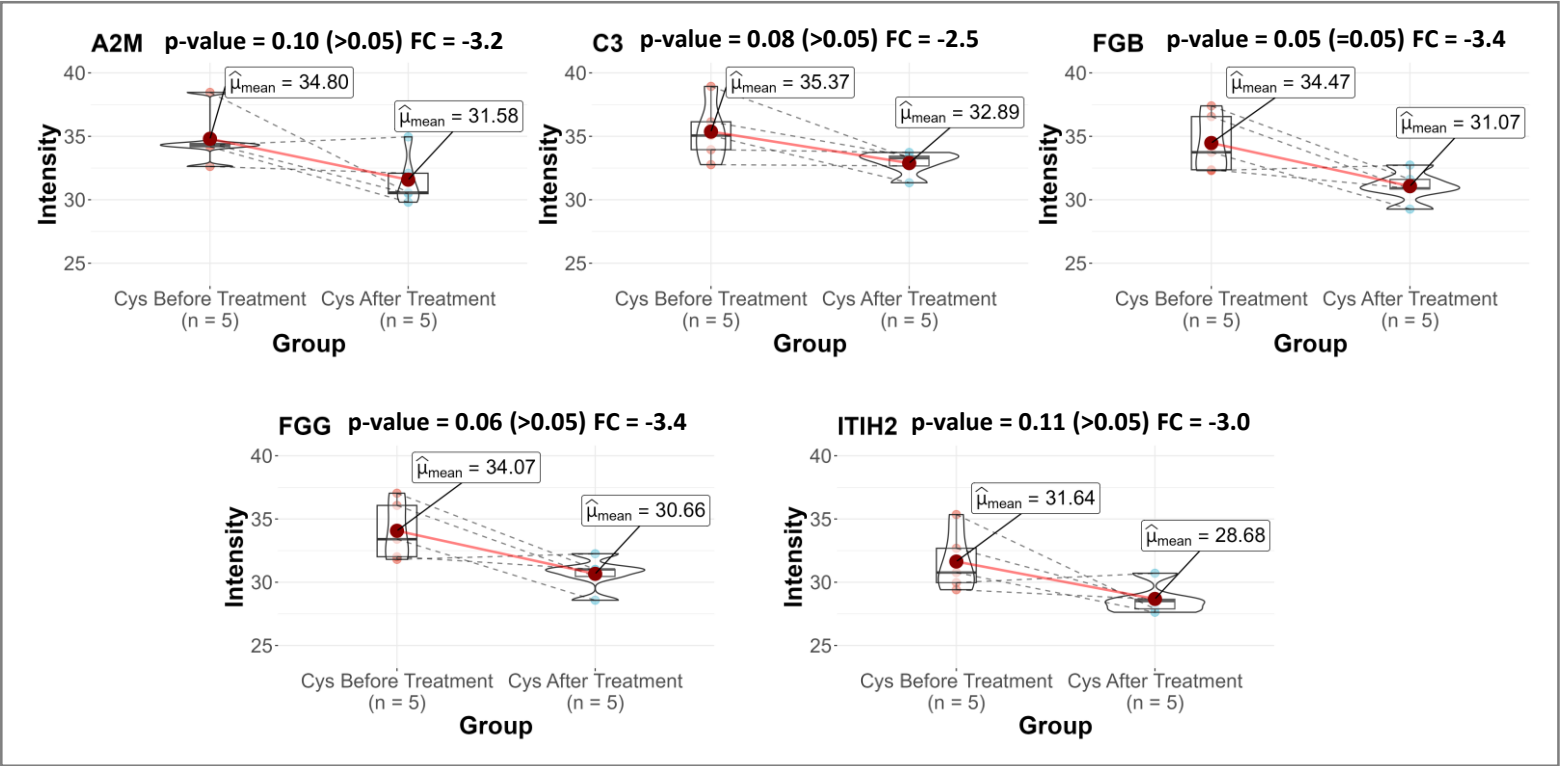

Supplement: sfae040_Supplemental_File [file sfae040_supplemental_file.zip › MaCO_Fig_S3_panelB.pdf]
